# Optimized training for jumping performance using the force-velocity imbalance: Individual adaptation kinetics

**DOI:** 10.1371/journal.pone.0216681

**Published:** 2019-05-15

**Authors:** Pedro Jiménez-Reyes, Pierre Samozino, Jean-Benoît Morin

**Affiliations:** 1 Centre for Sport Studies, Rey Juan Carlos University, Madrid, Spain; 2 Univ Savoie Mont Blanc, Laboratoire Interuniversitaire de Biologie de la Motricité, Chambéry, France; 3 Université Côte d’Azur, LAMHESS, Nice, France; 4 SPRINZ, Auckland University of Technology, Auckland, New Zealand; James Cook University College of Healthcare Sciences, BRAZIL

## Abstract

**Aims:**

We analysed the changes in force-velocity-power variables and jump performance in response to an individualized training program based on the force-velocity imbalance (FV_*imb*_). In particular, we investigated (i) the individual adaptation kinetics to reach the optimal profile and (ii) de-training kinetics over the three weeks following the end of the training program.

**Methods:**

Sixty subjects were assigned to four sub-groups according to their initial FV_*imb*_: high or low force-deficit (FD) and high or low velocity-deficit (VD). The duration of training intervention was set so that each individual reached their “Optimal force-velocity (F-v) profile”. Mechanical and performance variables were measured every 3 weeks during the program, and every week after the end of the individualized program.

**Results:**

All subjects in the FD sub-groups showed extremely large increases in maximal theoretical force output (+30±16.6% Mean±SD; ES = 2.23±0.28), FV_*imb*_ reduction (-74.3±54.7%; ES = 2.17±0.27) and large increases in jump height (+12.4±7.6%; ES = 1.45±0.23). For the VD sub-groups, we observed moderate to extremely large increases in maximal theoretical velocity (+15.8±5.1%; ES = 2.72±0.29), FV_*imb*_ reduction (-19.2±6.9%; ES = 2.36±0.35) and increases in jump height (+10.1±2.7%; ES = 0.93±0.09). The number of weeks needed to reach the optimal F-v profile (12.6 ± 4.6) was correlated to the magnitude of initial FV_*imb*_ (r = 0.82, p<0.01) for all participants regardless of their initial subgroup. No significant change in mechanical variables or jump performance was observed over the 3-week de-training period.

**Conclusions:**

Collectively, these results provide useful insights into a more specific, individualized (i.e. based on the type and magnitude of FV_*imb*_) and accurate training prescription for jumping performance. Considering both training content and training duration together with FV_*imb*_ may enable more individualized, specific and effective training monitoring and periodization.

## Introduction

The ability to perform ballistic muscle contractions during jumps, sprints or changes of direction determines performance in numerous sport activities and corresponds to the ability to reach the highest velocity in the shortest time with one´s own body mass. It is clearly determined by high levels of force, power, and the velocity produced during the push-off phase [1–4] and so is directly related to the mechanical properties of the neuromuscular system, and notably to power capabilities [5]. Recently, ballistic performance such as jumping has been shown to be largely determined by the maximal power output (*P*_*max*_) that the lower limbs can generate [6,7], but it is also influenced by the individual combination of the underlying capabilities to produce force at low and high velocities, known as the force-velocity (F-v) profile [5,8,9]. Thus, the measurement of individual F-v relationships and their contribution to ballistic performance may provide a more accurate and integrative mechanical representation of athletes’ maximal force production capabilities [5]. It is important since they encompass the entire F-v spectrum, from the theoretical maximal force that can be produced at null velocities (*F*_*0*_, force qualities) to the theoretical maximal velocity up to which force can be produced (*v*_*0*_, velocity qualities) [9]. This may lead to more individualized and effective training programs [9,10].

The “power-force-velocity profiling” approach is based on force- and power-velocity relationships characterizing the maximal mechanical capabilities of the lower limbs’ neuromuscular systems [9]. As shown theoretically [5,11] and confirmed experimentally [8], there is, for each individual, an optimal F-v profile that maximizes lower limb ballistic performance (e.g. vertical jumping) and represents the optimal balance between force and velocity qualities during jumping [5,8,12]. The relative difference between actual and optimal F-v profiles for a given individual represents the magnitude and the direction of the unfavorable balance between force and velocity qualities (i.e. the force-velocity imbalance, *FV*_*imb*_ in %), which allows individual determination of force or velocity deficit. The actual individual F-v profile and *P*_*max*_ can be determined from a series of 2 to 6 loaded vertical jumps [8,12–14], while the optimal F-v profile can be computed using Samozino et al.’s equations [5,8]. For a given *P*_*max*_, vertical jump performance has been shown to be negatively correlated to *FV*_*imb*_, which supports the importance of considering this individual characteristic in addition to *P*_*max*_ when designing training programs to improve ballistic performance [5,8,9].

For the F-v profile, it is worth noting that (i) an optimal F-v profile maximizing ballistic performance exists independently from *P*_*max*_ for each athlete; (ii) the F-v profile is related to specific strength training addressing the *FV*_*imb*_ [10]; and (iii) the F-v profile can differentiate between athletes and characterize their performance [15,16]. Quantifying *FV*_*imb*_ on an individual basis was recently shown to be an effective approach to training prescription, adapted to each athlete’s individual needs [10]. An interesting factor regarding the F-v profile when assessed during jumping is that the individual F-v profile responds to specific training corresponding to the various sections of the F-v spectrum, showing an improvement in ballistic performance through an effective shift in the individual F-v profile towards the optimal value (*FV*_*imb*_ reduction), and/or an increase in *P*_*max*_ [8,10]. For instance, when aiming to work on a force deficit, training should be focused on the force side of the F-v spectrum in order to increase *P*_*max*_ while decreasing *FV*_*imb*_. This can be done by increasing force production capabilities at low velocities (*F*_*0*_) as a priority [5]. In contrast, when the target is to work on a velocity deficit (at the other end of the F-v spectrum), the training should be aimed at increasing *P*_*max*_ by improving force production capabilities at high velocity (*v*_*0*_). Likewise, when aiming to work on the entire F-v spectrum, the training should focus on increasing *P*_*max*_ as a priority [2,17] while maintaining the F-v profile close to the optimal value (and thus *FV*_*imb*_ close to 0%).

On this basis, Jiménez-Reyes et al (2017) [10] suggested that specific strength training aimed at improving ballistic performance should be designed on an individual basis to both reduce *FV*_*imb*_ (i.e. to increase the *F*_*0*_ or *v*_*0*_ component of an individual’s F-v profile preferentially and shift it towards his/her optimal profile) and increase *P*_*max*._ This specific training was defined as “optimized training” or “individualized training based on *FV*_*imb*_” since the aim was to tailor the training prescription to the athlete’s individual F-v profile. Specifically, in this pilot study, participants were assigned to three training intervention groups (each of 9 weeks duration) based on their initial *FV*_*imb*_: (i) an optimized group divided into velocity-deficit, force-deficit and well-balanced sub-groups based on subjects’ *FV*_*imb*_; (ii) a “non-optimized” group for which the training program was not specifically based on *FV*_*imb*_; and (iii) a control group (with no specific training). This study showed that an optimized and individualized training program specifically addressing the *FV*_*imb*_ was more efficient for improving jumping performance than the traditional resistance training common to all subjects.

Despite being, to date, the only study on the effects of an optimized training program specifically addressing the *FV*_*imb*_ [10], this protocol had three main limitations. First, the fixed training duration of 9 weeks for all subjects was not ideal. Although the trends in the results were very clear and all the subjects in the optimized group responded as hypothesized, only some of them were close to the optimal profile at the end of the 9 weeks and a variability was observed that may have depended on several factors such as training background, time needed for adaptation, or the magnitude of the initial F-v deficit. Thus, it is reasonable to suggest that the duration of the program should *also* be individualized and be as long as necessary for *each* individual to reach an *FV*_*imb*_ close to 0 (considering that an optimal F-v profile is regarded as ± 10% of *FV*_*imb*_). Secondly, in the previous study, all subjects improved their jump height and reduced *FV*_*imb*_, but the level of improvement varied when comparing the velocity and force deficit sub-groups; the velocity-deficit sub-group tended to almost reach the optimal profile in the fixed 9-week training period, while the force-deficit sub-group were not as close to the optimal profile. It is likely that the time required for adjustments at a structural level (mainly related to *F*_*0*_) is longer [18] than that required for more acute neuromuscular adaptations (more related to both *F*_*0*_ and *v*_*0*_). The next step for complete individualization would thus be to consider not only training content but also training duration, and account for individual training response kinetics. This complete and dynamic individualization would provide the possibility of modifying training for those subjects who adapted faster than others and to allow changes in sub-groups during the training periods to finely adapt the response kinetics of each individual. Finally, Jiménez-Reyes et al (2017) [10] studied training adaptation, but did not consider what would happen after the training period, i.e. how individual *FV*_*imb*_ values changed after specific training cessation. This question is crucial, since it is not known whether a significant decrease in *FV*_*imb*_ will be sustained (and if so, for how long) or reversed (and if so, how fast). This information may help better design and periodise specific training for ballistic performance using the force-velocity imbalance approach both in individual and team sports, for example during taper periods or training camps preparing for major competitions. Considering the need for replication in scientific studies, and the limitations and unknowns discussed above, we decided that a “replication” study with an improved design was warranted.

The aims of this study were (i) to analyze the individual adaptation kinetics in force-velocity-power profile until every subject reached their optimal profile, and study the associated training duration variability and adapt subjects’ training content during the protocol in case of changes in deficit categories; and (ii) to study the individual kinetics of de-training over the three weeks following the training program. We hypothesized that, as observed by Jiménez-Reyes et al (2017) [10], the individualized training content based on *FV*_*imb*_ would induce clear improvements in jump height, and that the more complete individualization (in terms of timing and training content) would lead to even more systematic and clear improvements in performance. In addition, based on our previous results, we hypothesized that, all other things being equal, force-deficit individuals would need more time to reach their optimal profile than velocity-deficit ones. Finally, since the detraining aspects of this study were novel, no specific hypothesis could be formulated for this part of the study.

## Materials and methods

### Subjects

Sixty trained athletes (age = 23.7 ± 3.7 years, body mass = 76.4 ± 9.3 kg, height = 1.79 ± 0.05 m, SJ = 0.32 ± 0.03 m) gave their written informed consent to participate in this study, which was approved by the local ethics committee of the Catholic University of San Antonio (Murcia) in agreement with the Declaration of Helsinki. All subjects were professional futsal or semi-professional soccer and rugby players. All athletes had a strength-training background of at least one year, were highly trained (average weekly training volume of 12 hours at the time of the study), and familiar with the testing procedures.

The present study used a longitudinal follow-up with pre-post design with testing sessions before reaching the optimal F-v profile according to the percentage thresholds of *FV*_*imb*_. All tests were conducted at the same time of day, from 17:00 to 21:00. Each subject underwent anthropometric assessment and performed loaded squat jumps (SJ) to determine the individual F-v relationships, *P*_*max*_ values and *FV*_*imb*_ (see next section). *FV*_*imb*_ was then used as the reference to assign participants to different training groups and sub-groups at the beginning of the intervention. Since the hypothesis was that performance improvement would result from increasing *P*_*max*_ and/or decreasing *FV*_*imb*_ [9], and because of the previous work based on *FV*_*imb*_ [10], *FV*_*imb*_ was the criterion used for designing individualized training programs in this study.

### Testing procedure and data processing

#### F-v relationships of the lower limb neuromuscular system in Squat Jump (SJ)

To determine individual F-v relationships, each subject performed vertical maximal SJs without loads and against five to eight extra loads ranging from 15 to 90 kg in a randomized order. The test was performed on a Smith machine (Multipower Fitness Line, Peroga, Spain) that allowed a smooth vertical displacement of the bar along a fixed vertical path. Before each SJ condition with no additional load, participants were instructed to stand up straight and still at the center of the jumping area. They kept their hands on their hips for jumps without load and on the bar for loaded jumps, this hand position remaining the same during the entire movement. Subjects were asked to maintain their individual starting position (approximately 90° knee angle) for about 2 s and then apply force as fast as possible and jump for maximum height. No countermovement was allowed and was visually checked. If all these requirements were not met, the trial was repeated. Two valid trials were performed with each load with two minutes of recovery between trials and four to five minutes between load conditions.

Mean mechanical parameters were calculated for each loading condition using Samozino’s method [11], based on Newton’s second law of motion. This method establishes that mean force (*F*), velocity (*v*), and power (*P*) can be calculated during a vertical jump from measurement of the jump height and squat jump positions. Jump height was obtained using an OptoJump optical measurement system (Microgate, Bolzano, Italy). Force, velocity and power were calculated using three equations considering only simple input variables: body mass, jump height and push-off distance. The latter corresponds to the distance covered by the center of mass during push-off, i.e. the extension range of the lower limbs from the starting position to take-off [11], and was measured a priori for each subject as the difference between the extended lower limb length (iliac crest to toes with plantar flexed ankle) and the length in the individual standardized starting position (iliac crest to ground vertical distance).

F-v linear relationships were determined using the best trials from each loading condition and least squares linear regressions. F-v curves were extrapolated to obtain *F*_*0*_ (then normalized to body mass) and *v*_*0*_, which respectively correspond to the intercepts of the F-v curve with the force and velocity axis. The F-v profile, which is the slope of the F-v linear relationship, was then computed from *F*_*0*_ and *v*_*0*_ according to Samozino et al (2012) [5]. Values of *P*_*max*_ (normalized to body mass) were determined as: *P*_*max*_ = *F*_*0*_· *v*_*0*_/4 [5,6,8]. From *P*_*max*_ and push-off distance values, an individual theoretical optimal F-v profile (normalized to body mass, in N.s.kg^-1^.m^-1^), maximizing vertical jumping performance, was computed for each subject using equations proposed by Samozino et al (2012) [5]. The F-v imbalance (*FV*_*imb*_, in %), was then individually computed as recently proposed by Samozino et al (2014) [8]

$${Fv}_{imb}=100.|1-\frac{S_{Fv}}{S_{Fv}opt}|$$ (1)

An *FV*_*imb*_ value around 0% indicates an F-v profile equal to 100% of the optimal profile (perfect balance between force and velocity qualities), whereas an F-v profile value higher or lower than the optimal indicates a profile too heavily oriented towards force or velocity capabilities, respectively. The reliability of these variables and approaches has been shown previously (for details, see [8,11,12,14]).

### Experimental design

After initial testing of their individual F-v properties, participants were assigned to Force Deficit (FD) or Velocity Deficit (VD) groups, and within each group to a high force deficit (HFD) sub-group (n = 18; body mass = 74.0 ± 7.9 kg, height = 1.79 ± 0.06 m, SJ = 0.31 ± 0.03 m); a high velocity deficit (HVD) sub-group (n = 10; body mass = 83.8 ± 9.0 kg, height = 1.81 ± 0.03 m, SJ = 0.33 ± 0.02 m), a low force deficit (LFD) sub-group (n = 18; body mass = 71.9 ± 8.8 kg, height = 1.79 ± 0.06 m, SJ = 0.32 ± 0.03 m); and a low velocity deficit (LVD) sub-group (n = 14; body mass = 80.0 ± 7.7 kg, height = 1.79 ± 0.05 m, SJ = 0.34 ± 0.03 m). The training program was adjusted for the participants in each group according to their *FV*_*imb*_. The training program was slightly different with regard to intensity and similar in volume, although the exercises were generally familiar for almost all participants. Training intervention was performed in the middle of the competitive season for all participants.

During the training period, each group followed a training intervention according to the *FV*_*imb*_ threshold and the ratios of work proposed by Jiménez-Reyes et al (2017) [10], focusing on different sections of the F-v spectrum taking into account the needs of the athletes. For instance, the HFD sub-group performed mainly force-oriented (very high load) training, while the HVD sub-group performed velocity-oriented (ballistic, with very high velocity of limb extension) training. The LFD and LVD subgroups undertook similar training, but with a shift to the center of the F-v spectrum. The training features, according to the *FV*_*imb*_ threshold, are detailed in Jiménez-Reyes et al [10]. The duration of training intervention was not fixed beforehand but was established as the duration necessary for each individual to reach an *FV*_*imb*_ close to 0 (an “optimal F-v profile” was accepted for values of *FV*_*imb*_ of ± 10%, corresponding to the “well-balanced” category threshold proposed by Jiménez-Reyes et al (2017) [10]. During training interventions, the F-v profile was measured every 3 weeks, monitoring all F-v profile variables and *FV*_*imb*_. When subjects reached a new *FV*_*imb*_ threshold they changed training group and thus training content according to the new threshold. Finally, when athletes were very close to their optimal F-v profile, the F-v profile was monitored every 2 weeks when they were within 5–10% of 90% (LFD) or 110% (LVD) and then every week when they were within 0–5% of 90% (LFD) or 110% (LVD). This frequent monitoring allowed us to accurately determine the exact time needed to reach the optimal F-v profile. Once subjects reached their optimal F-v profile they stopped the specific training targeted to reduce *FV*_*imb*_ (but not their usual sport practice, which was continued as during the experimental phase). During the study intervention, all players performed their usual sport-specific training (e.g. technical, tactical, small sided games) with similar volume and specific training, which was carefully controlled. During the following 3-week period (subjects voluntarily refrained from strength training for 3 weeks while continuing with their specific sporting activities and competitions) we studied the potential de-training process by monitoring the F-v profile variables each week.

### Training intervention

Considering the aforementioned elements of the specificity of training to improve either the maximal force or velocity aspects of the F-v spectrum (e.g. [17,19–26]), the HFD, LFD, HVD and LVD training groups were established according to individuals’ *FV*_*imb*_. For each one of these sub-groups, we considered not only the type of deficit (either in force or in velocity), but also its magnitude. Therefore, in each sub-group, the training program was established according to specific *FV*_*imb*_ thresholds, as detailed in Table 1, (Jiménez-Reyes et al) [10].

**Table 1:** Force-velocity imbalance categories, thresholds and associated resistance training load ratios together with loading target for the F-v spectrum and exercises and training loads for each exercise.

*FV*_*imb*_ *Categories*	*F-v Profile in % of OPTIMAL* *Thresholds (%)*	Training loads ratio*	*Loading focus/target*	*Exercises*	Training loads
		3 Strength		Back Squat	80–90% 1RM
High Force Deficit	<60	2 Strength-Power	Strength	Leg Press	90–95% 1RM
		1 Power		Deadlift	90–95% 1RM
		2 Strength		Clean Pull	80% 1RM
Low Force Deficit	60–90	2 Strength-Power	Strength-Power	Deadlift	80% 1RM
		2 Power		SJ	> 70% of BW
		1 Strength		CMJ	> 80% of BW
		1 Strength-Power		SJ	20–30% of BW
Well-Balanced	> 90–110	2 Power	Power	CMJ	35–45% of BW
		1 Power-Speed		Single leg SJ	BW
		1 Speed		Single leg CMJ	10% of BW
				Clean Pull Jump	65% 1 RM
		2 Speed		Depth Jumps	
Low Velocity Deficit	> 110–140	2 Power-Speed	Power-Speed	SJ	BW
		2 Power		CMJ	10% of BW
				Maximal VBJ	
		3 Speed			
High Velocity Deficit	> 140	2 Power-Speed	Speed	Horizontal SJ	< BW
		1 Power		CMJ with arms	BW

Abbreviations: *FV*_*imb*_, F-v imbalance; RM, repetition maximum; SJ, Squat Jump; BW, body weight; CMJ, Countermovement Jump; VBJ, Vertical Box Jump.

* Ratio based on six exercises/wk, three sets/exercise and 18 sets/wk.

According to previous findings showing improvements in maximal strength, power and ballistic performance after specific training (e.g. [17,20]), the individualized training programs proposed here involved maximal effort and were mainly designed by setting the loads to vary the movement velocity, and in turn to target different parts of the F-v curve. For example, “Strength” exercises used high loads of ~ *F*_*0*_ moved at low velocity, such as >80% of one repetition maximum in back squat, whereas “Speed” exercises used a force of ~body mass moved at high velocity, enhanced using exercises inducing a lower limb extension velocity beyond that of a squat jump, using the stretch-shortening cycle (e.g. CMJ) or assisted/low resistance push-offs (e.g. band-assisted SJ or horizontal-assisted roller) [27]. For more details see Table 1.

### Statistical analysis

All data are presented as mean ± SD. In order to clearly assess the practical meaning of the results, data were analysed using the magnitude-based inference approach [28].

Within-group differences in pre- and post-training jump height, F-v profile in (%) of optimal F-v, *F*_*0*_ and *v*_*0*_ were assessed using standardised effect sizes (ES). The magnitudes of the within-group changes were interpreted using values of trivial (< 0.20), small (0.20 –< 0.60), moderate (0.60 –< 1.20), large (1.20 –< 2.00) and extremely large (> 2.00) for the between-athlete variation at pre (i.e. smallest worthwhile change).

The probability that these differences actually existed was then assessed via magnitude-based qualitative inference [29]. Qualitative inferences were based on the quantitative chances of benefit outlined by Hopkins et al (2009) [28]. Clinical chances are the percentage chances that an observed effect is clinically positive/trivial/negative; e.g. (40/40/20%) means an effect has a 40% chance of being positive, a 40% chance of being trivial and a 20% chance of being negative. Probabilities that differences were higher than, lower than, or similar to the smallest worthwhile difference were evaluated qualitatively as: possibly, 25% to 74.9%; likely, 75% to 94.9%; very likely, 95% to 99.5%; and most (extremely) likely, >99.5%.

A stepwise multiple regression analysis was also performed to test the association between individual *FV*_*imb*_ and *P*_*max*_ changes (independent variables) with jump height changes (dependent variable).

## Results

Mean ± SD values for all performance and mechanical variables pre and post training intervention and for the 3-week period of de-training, obtained by monitoring F-v profiles each week, are shown for all groups and sub-groups in Tables 2 and 3, along with the qualitative inferences for within-group changes. During the de-training period all the parameters maintained their post-training values with minimal differences.

**Table 2:** Changes in variables associated to Force-velocity profile in different sub-groups.

	Pre	Opt	Weeks	Post ‒ Pre			
	$\bar{x}$ ± SD	$\bar{x}$ ± SD	$\bar{x}$ ± SD	%Δ ± SD	*ES; ±90% CL*	*Inference and Probability*	
**F-v (%) Optimal F-v**							
*Force Deficit*	56.4 ± 15.4	90.5 ± 0.8	12.6 ± 4.6	74.3 ± 54.7	2.17 ± 0.27	***Ext*. *Large*** ↑	*most likely*
*HFD*	43.1 ± 8.6	90.4 ± 0.7	15.9 ± 3.8	118.1 ± 46.5	5.27 ± 0.39	***Ext*. *Large*** ↑	*most likely*
*LFD*	69.6 ± 6.5	90.6 ± 0.8	9.2 ± 2.0	31.1 ± 11.7	3.09 ± 0.38	***Ext*. *Large*** ↑	*most likely*
*Velocity Deficit*	135.5 ± 10.9	108.8 ± 1.3	8.7 ± 2.1	-19.2 ± 6.9	-2.36 ± 0.35	***Ext*. *Large*** ↓	*most likely*
*HVD*	146.1 ± 4.8	108.7 ± 1.3	9.6 ± 1.9	-25.5 ± 2.4	-7.19 ± 0.52	***Ext*. *Large*** ↓	*most likely*
*LVD*	128.0 ± 7.1	108.9 ± 1.4	8.0 ± 2.1	-14.6 ± 5.3	-2.54 ± 0.15	***Ext*. *Large*** ↓	*most likely*
***P***_**max**_ **(W·kg**^**-1**^**)**							
*Force Deficit*	27.0 ± 3.4	27.0 ± 2.9		0.44 ± 6.35	0.00 ± 0.15	***Trivial***	*very likely*
*HFD*	28.5 ± 3.7	27.6 ± 3.7		-2.99 ± 7.19	-0.24 ± 0.21	***Small*** ↓	*possibly*
*LFD*	25.5 ± 2.1	26.4 ± 1.8		3.87 ± 2.54	0.44 ± 0.12	***Small*** ↑	*most likely*
*Velocity Deficit*	24.8 ± 3.3	26.7 ± 3.7		7.79 ± 2.23	0.56 ± 0.06	***Small*** ↑	*most likely*
*HVD*	25.9 ± 2.7	27.9 ± 3.0		20.2 ± 2.41	0.68 ± 0.15	***Moderate*** ↑	*most likely*
*LVD*	24.0 ± 3.6	25.9 ± 4.0		7.87 ± 1.81	0.49 ± 0.07	***Moderate*** ↑	*most likely*
***F***_**0**_ **(N·kg**^**-1**^**)**							
*Force Deficit*	30.2 ± 3.8	38.9 ± 3.0		30.3 ± 16.6	2.23 ± 0.28	***Ext*. *Large*** ↑	*most likely*
*HFD*	27.6 ± 3.5	39.5 ± 3.7		44.1 ± 11.7	3.24 ± 0.29	***Ext*. *Large*** ↑	*most likely*
*LFD*	32.9 ± 1.7	38.3 ± 2.0		16.5 ± 5.14	3.00 ± 0.36	***Ext*. *Large*** ↑	*most likely*
*Velocity Deficit*	44.7 ± 4.4	41.6 ± 4.0		-6.77 ± 4.08	-0.68 ± 0.15	***Moderate*** ↓	*most likely*
*HVD*	47.3 ± 2.4	42.4 ± 2.8		-10.4 ± 2.04	-1.85 ± 0.19	***Large*** ↓	*most likely*
*LVD*	42.8 ± 4.5	41.1 ± 4.8		-4.15 ± 2.96	-0.37 ± 0.13	***Small*** ↓	*very likely*
***v***_**0**_ (m·s^-1^)							
*Force Deficit*	3.63 ± 0.69	2.77 ± 0.11		-21.4 ± 12.7	-1.22 ± 0.27	***Large*** ↓	*most likely*
*HFD*	4.17 ± 0.59	2.78 ± 0.13		-32.1 ± 8.5	-2.25 ± 0.37	***Ext*. *Large*** ↓	*most likely*
*LFD*	3.10 ± 0.20	2.76 ± 0.07		-10.7 ± 4.3	-1.61 ± 0.31	***Large*** ↓	*most likely*
*Velocity Deficit*	2.21 ± 0.12	2.56 ± 0.14		15.8 ± 5.1	2.72 ± 0.29	***Ext*. *Large*** ↑	*most likely*
*HVD*	2.18 ± 0.12	2.62 ± 0.12		20.2 ± 2.4	3.30 ± 0.18	***Ext*. *Large*** ↑	*most likely*
*LVD*	2.24 ± 0.12	2.52 ± 0.13		12.6 ± 3.9	2.13 ± 0.29	***Ext*. *Large*** ↑	*most likely*
***Jump Height* (m)**							
*Force Deficit*	0.32 ± 0.03	0.36 ± 0.03		12.5 ± 7.6	1.45 ± 0.23	***Large*** ↑	*most likely*
*HFD*	0.31 ± 0.03	0.36 ± 0.04		17.1 ± 8.1	1.76 ± 0.33	***Large*** ↑	*most likely*
*LFD*	0.33 ± 0.02	0.35 ± 0.02		7.8 ± 2.8	1.27 ± 0.17	***Large*** ↑	*most likely*
*Velocity Deficit*	0.33 ± 0.03	0.36 ± 0.04		10.1 ± 2.7	0.93 ± 0.09	***Moderate*** ↑	*most likely*
*HVD*	0.34 ± 0.02	0.36 ± 0.03		11.6 ± 2.8	1.12 ± 0.13	***Moderate*** ↑	*most likely*
*LVD*	0.32 ± 0.03	0.35 ± 0.04		9.1 ± 2.2	0.78 ± 0.09	***Moderate*** ↑	*most likely*

Values are mean ± standard deviation, percent change ± standard deviation and standardised effect size; ±90% confidence limits. Abbreviations: $\bar{x}$, mean; SD, standard deviation, %Δ, percent change; ES, effect size; 90% CL, 90% confidence limits; Ext, extremely; ↑, positive effect; ↓, negative effect; *P*_max_, maximal power output; W, watt; kg, kilogramme; *F*_0_, theoretical maximal force; N, newton; *v*_0_, theoretical maximal velocity; m, metre; s, second; Opt, moment at each individual reach a FV_imb_ close to 0 (considering that an “Optimal F-v profile” was accepted for values of FV_imb_ of ± 10%, which corresponds to the “Well-balanced” category. Qualitative inferences are trivial (< 0.20), small (0.20 –< 0.60), moderate (0.60 –< 1.20), large (1.20 –< 2.00) and extremely large (> 2.00): possibly, 25 –< 75; likely, 75 –< 95%; very likely, 95 –< 99.5%; most likely, > 99.5. Note: weeks to Optimal FV profile were the same than the first variable for all the variables.

**Table 3:** Changes in variables associated to Force-velocity profile in different sub-groups and detraining effects.

			Training	Detraining		
			OPT	WEEK 1	WEEK 2	WEEK 3
			$\bar{x}$ ± SD	$\bar{x}$ ± SD	$\bar{x}$ ± SD	$\bar{x}$ ± SD
***FD***		*F*_0_ (N·kg^-1^)	38.9 ± 3.0	38.6 ± 2.9	38.4 ± 2.9	38.1 ± 3.0
		*v*_0_ (m·s^-1^)	2.77 ± 0.11	2.80 ± 0.11	2.80 ± 0.11	2.82 ± 0.11
		*P*_max_ (W·kg^-1^)	27.0 ± 2.9	27.1 ± 3.0	26.9 ± 3.0	26.8 ± 2.9
		FV_IMB_ (%)	90.5 ± 0.8	88.8 ± 1.2	88.2 ± 1.2	87.2 ± 1.6
		*SJ* (m)	0.36 ± 0.03	0.36 ± 0.03	0.35 ± 0.03	0.35 ± 0.03
	*HFD*	*F*_0_ (N·kg^-1^)	39.5 ± 3.7	39.2 ± 3.7	38.9 ± 3.7	38.5 ± 3.8
		*v*_0_ (m·s^-1^)	2.78 ± 0.13	2.82 ± 0.13	2.82 ± 0.13	2.84 ± 0.13
		*P*_max_ (W·kg^-1^)	27.6 ± 3.7	27.7 ± 3.7	27.5 ± 3.8	27.4 ± 3.7
		FV_IMB_ (%)	90.4 ± 0.7	88.4 ± 0.8	87.8 ± 0.9	86.3 ± 1.3
		*SJ* (m)	0.36 ± 0.04	0.36 ± 0.04	0.36 ± 0.04	0.36 ± 0.04
	*LFD*	*F*_0_ (N·kg^-1^)	38.3 ± 2.0	38.1 ± 1.9	37.8 ± 1.9	37.6 ± 1.8
		*v*_0_ (m·s^-1^)	2.76 ± 0.07	2.79 ± 0.08	2.79 ± 0.08	2.79 ± 0.09
		*P*_max_ (W·kg^-1^)	26.4 ± 1.8	26.5 ± 1.8	26.4 ± 1.7	26.3 ± 1.7
		FV_IMB_ (%)	90.6 ± 0.8	89.3 ± 1.4	88.6 ± 1.2	88.1 ± 1.4
		*SJ* (m)	0.35 ± 0.02	0.35 ± 0.02	0.35 ± 0.02	0.35 ± 0.02
***VD***		*F*_0_ (N·kg^-1^)	41.6 ± 4.1	41.6 ± 4.0	41.5 ± 4.0	41.4 ± 4.0
		*v*_0_ (m·s^-1^)	2.56 ± 0.14	2.56 ± 0.14	2.55 ± 0.14	2.54 ± 0.14
		*P*_max_ (W·kg^-1^)	26.7 ± 3.7	26.7 ± 3.7	26.5 ± 3.7	26.4 ± 3.7
		FV_IMB_ (%)	108.8 ± 1.3	109.1 ± 1.4	109.2 ± 1.5	109.2 ± 1.5
		*SJ* (m)	0.36 ± 0.04	0.36 ± 0.04	0.36 ± 0.04	0.36 ± 0.04
	*HVD*	*F*_0_ (N·kg^-1^)	42.4 ± 2.8	42.3 ± 2.7	42.2 ± 2.7	42.0 ± 2.7
		*v*_0_ (m·s^-1^)	2.62 ± 0.12	2.63 ± 0.13	2.62 ± 0.12	2.62 ± 0.12
		*P*_max_ (W·kg^-1^)	27.9 ± 3.0	27.9 ± 3.0	27.7 ± 3.0	27.6 ± 2.9
		FV_IMB_ (%)	108.7 ± 1.3	108.5 ± 1.5	108.3 ± 1.5	108.1 ± 1.4
		*SJ* (m)	0.38 ± 0.03	0.38 ± 0.03	0.38 ± 0.03	0.38 ± 0.03
	*LVD*	*F*_0_ (N·kg^-1^)	41.1 ± 4.8	41.2 ± 4.7	41.1 ± 4.7	41.0 ± 4.7
		*v*_0_ (m·s^-1^)	2.52 ± 0.13	2.51 ± 0.13	2.49 ± 0.13	2.49 ± 0.13
		*P*_max_ (W·kg^-1^)	25.9 ± 4.0	25.9 ± 4.0	25.7 ± 4.0	25.6 ± 4.0
		FV_IMB_ (%)	108.9 ± 1.4	109.5 ± 1.1	109.9 ± 1.1	109.9 ± 0.9
		*SJ* (m)	0.35 ± 0.04	0.35 ± 0.04	0.35 ± 0.04	0.35 ± 0.04

Values are mean ± standard deviation. Abbreviations: $\bar{x}$, mean; SD, standard deviation; *P*_max_, maximal power output; W, watt; kg, kilogramme; OPT, moment at each individual reach a FV_imb_ close to 0 (considering that an “Optimal F-v profile” was accepted for values of FVimb of ± 10%, which corresponds to the “Well-balanced” category); *F*_0_, theoretical maximal force; N, newton; *v*_0_, theoretical maximal velocity; FV_imb_, Force-velocity imbalance; m, metre; s, second; HFD, High Force Deficit Sub-group; HVD, High Velocity Deficit Sub-group; LFD, Low Force Deficit Sub-group; LVD, Low Velocity Deficit Sub-group.

**Fig 1:**
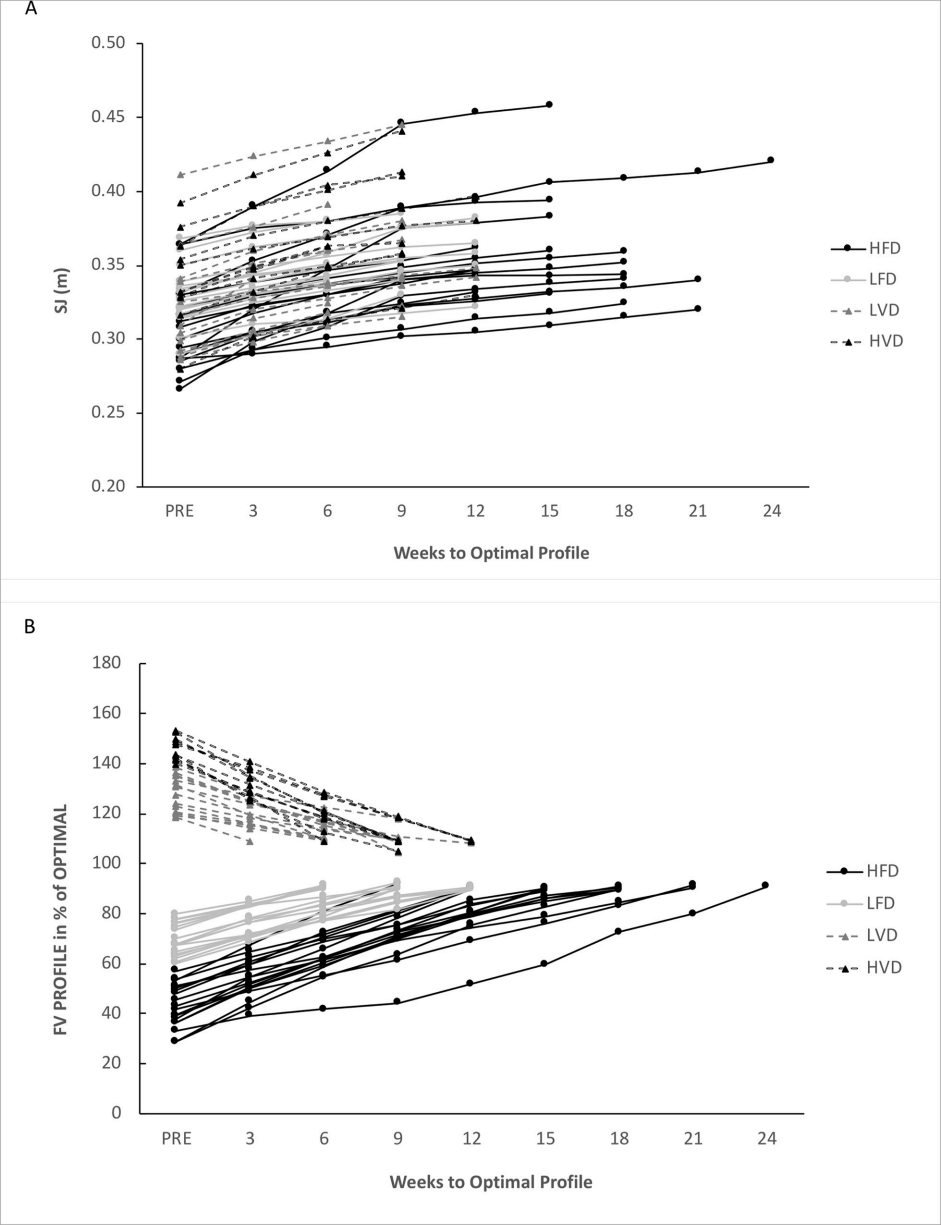
**A-B:** Individual changes in jump height (A) and *FV*_*imb*_ (B) according to training weeks for each sub-group until they reached their optimal F-v profile.

**Fig 2:**
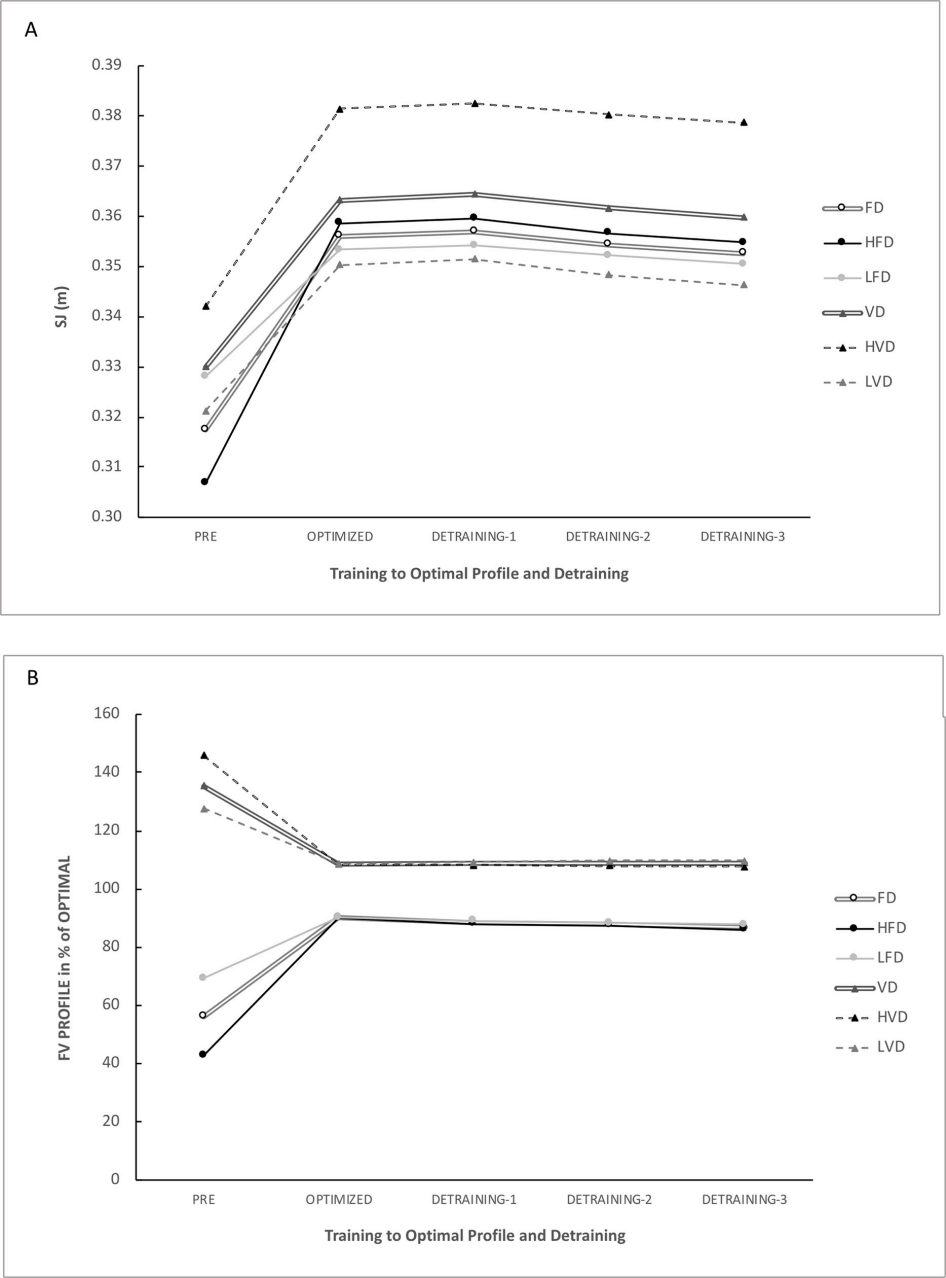
**A-B:** changes in jump height (A) and *FV*_*imb*_ (B) during training and de-training periods for each sub-group.

The FD and VD groups represent the averaged values obtained for both the HFD and LFD sub-groups (for the FD group) and the averaged values obtained for both the HVD and LVD sub-groups (for the VD group). The FD and VD groups and all their sub-groups showed extremely large changes in *FV*_*imb*_, together with an extremely large change in *F*_*0*_ (for the FD group) and *v*_*0*_ (for the VD group), respectively (Table 2; Fig 1. Additionally, substantial improvements in jump performance were observed in the FD and VD groups and associated sub-groups (+9.1 to +17.1% on average, most likely with moderate to large effects) (Fig 2).

**Fig 3:**
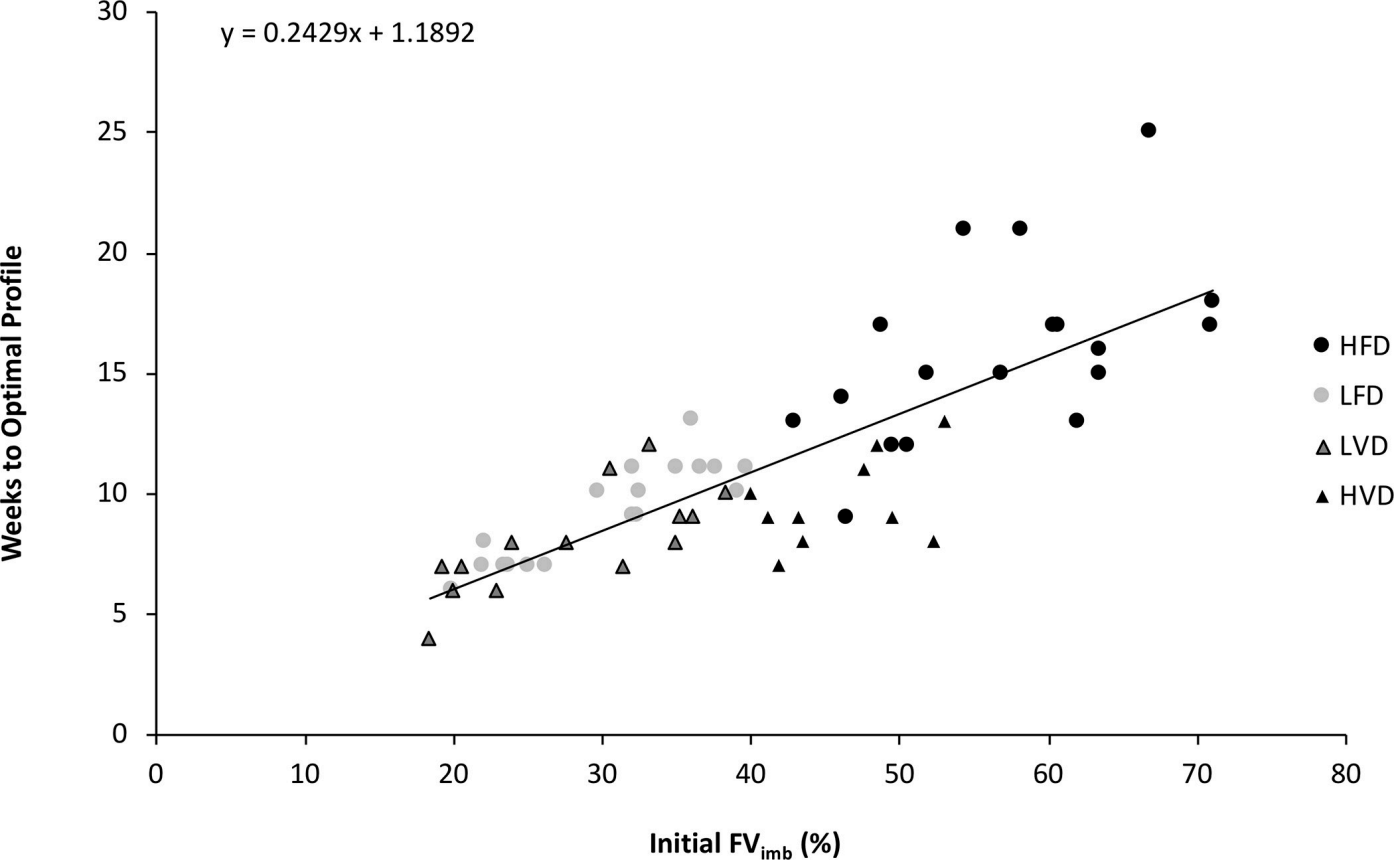
Correlation between initial *FV*_*imb*_ and time needed to reach an optimal F-v profile.

Fig 3 shows that there was a significant correlation between the number of weeks needed to reach the optimal FV profile and the initial *FV*_*imb*_ (r = 0.82 (0.74–0.89), p<0.01) for all individual participants. In the initial subgroups, the correlations were: LFD (r = 0.88, p<0.01); HFD (r = 0.54, p<0.05), LVD (r = 0.73, p<0.01); and HVD (r = 0.45, p = 0.183), respectively.

Stepwise multiple regression analysis showed that inter-individual differences in SJ height changes were significantly associated with differences in both *FV*_*imb*_ (explained variance of 48.2%, P<0.001) and *P*_*max*_ (explained variance of 37.7%, P<0.001). The quality of the final model (RMSE and R^2^), as well as the raw and standardized coefficients, are presented in Table 4.

**Table 4:** Stepwise multiple regression analysis for association between individual FV_imb_ and P_max_ changes (independent variables) with jump height changes (dependent variable).

*Multiple* *Regression* *Model*		*Adjusted R*^*2*^	RMSE		*Unstandardized* *coefficient*	*Standard* *Error*	Standardized coefficient	t	L
	1	0.482	<0.001	Intercept	7.051	0.831		8.487	<0.001
				FV_imb__PRE-POST	0.085	0.011	0.701	7.476	<0.001
	2	0.959	<0.001	Intercept	-2.896	0.45		-6.435	<0.001
				FV_imb__PRE-POST	0.2	0.005	1.645	36.511	<0.001
				P_max__PRE-POST	1.157	0.045	1.167	25.897	<0.001

Abbreviations: RMSE: Root Mean Square Error; FVIMB_ PRE-POST: Changes in FV_imb_ after training intervention; P_max__PRE-POST: changes in maximal power output in FV profile.

## Discussion

The main findings of this study confirmed the results of Jiménez-Reyes et al [10] that an optimized and individualized training program specifically addressing *FV*_*imb*_ is an effective strategy for improving jumping performance when controlling the time to reach an optimal F-v profile. This study also showed that setting the training duration and program content according to specific individual changes in F-v profile allowed each individual to eventually reach their optimal profile. Finally, this study was the first to test changes in *FV*_*imb*_ after stopping the specific training once an optimal F-v profile was reached. The results show that the training-induced adaptations remained unchanged overall during the 3-week period following the cessation of specific, individualized training.

Overall, optimized training aimed at reducing *FV*_*imb*_ and improving jump height provided beneficial effects in a range of performance variables related to the F-v relationship, including *F*_*0*_, *v*_*0*_, *P*_*max*_, *FV*_*imb*_ and jump height (Tables 1 and 2). The magnitude of changes observed ranged from small to extremely large with *possibly* to *most likely* probabilities. To date, only one study has tested the training effect of such a training program [10]. The novel aspect of the present study was to consider a dynamic approach to the duration of the program, in contrast to the fixed-time approach common to all subjects in our previous study [10]. With this approach, athletes followed the training program (tailored to their individual needs as indicated by their *FV*_*imb*_) until every individual reached their optimal *FV*_*imb*_ (i.e. less than 10% absolute value). This was shown to be a more adequate, complete and dynamic individualization than in our previous study since regardless of whether athletes were fast or slow responders to the specific training program, both the training content and duration were regulated to elicit target adaptations. Overall our results suggest that this dynamic individualized approach produced marked improvements in terms of training effectiveness (on an individual and group basis) compared to fixed-time and pre-set program durations, which are common approaches in strength and conditioning research and practice. The present study also showed that the training-induced change in SJ height was related to both *FV*_*imb*_ (48.2% of explained variance in jump height changes) and *P*_*max*_ (37.7% of the explained variance) changes. Interestingly, *FV*_*imb*_ changes explained a greater part of the inter-subject differences in jump performance changes than *P*_*max*_ changes, and *FV*_*imb*_ changes had a greater effect on performance change than *P*_*max*_ changes (standardized coefficients of 1.65 and 1.17, respectively).

Traditionally, a “one-size-fits-all” approach has been used to develop specific strength training programs, and overall positive effects have been reported for different programs focusing on improving jump performance, despite inconsistencies in the training prescription: e.g. heavy loads for all subjects [20,21,24,25,30–35]; light loads [32,36–38]; or combined strength training [21,32,35,37,39–41]. There are important limitations to this approach: the training content was the same for all subjects without taking into account their initial needs in terms of physical capabilities, or their individual responses to training over the course of the program. This can induce great variability in program effectiveness and unclear overall group performance responses to training [20,21,25,32,33,36,37,42,43].

Considering the aforementioned and the potential of using an individualized training program specifically addressing the *FV*_*imb*_, Jiménez-Reyes et al [10] compared a traditional approach with resistance training, common to all subjects regardless of their F-v imbalance, and optimal F-v profile training based on an F-v approach specifically addressing the *FV*_*imb*_. The results demonstrated the effectiveness of optimized training versus a “one-size-fits-all” approach: improvement in jump height was only significant in the optimized training group, with all subjects responding positively above the smallest worthwhile change threshold, while there was a very high variability and even some negative responders in the traditional approach (“one-size-fits-all”).

Despite these results and improvements reported by Jiménez-Reyes et al [10], it should be noted that one important limitation in their study was the fixed training duration of 9 weeks for all subjects. This fixed duration was almost appropriate for most subjects with velocity deficits, who completed the training intervention close to their optimal F-v profile. However, it was not long enough for most subjects with force deficits. Considering this limitation, the current study included not only an individualized training program but *also* an individualized training duration; i.e. as long as necessary for each individual to reach an optimal *FV*_*imb*_. The very high variability in training duration observed here (4 to 25 weeks for those at the extremes) supports the need for such an approach, compared to fixed program durations (Fig 3).

In the present protocol, all the subjects tested were assigned to specific sub-groups (HFD, LFD, HVD and LVD) and then given a specific training program proposed by Jiménez-Reyes et al [10]. Each of these sub-groups is discussed separately.

### Force-deficit group–(HFD and LFD sub-groups)

For the FD group, the specific heavy-load program resulted in extremely large increases in *F*_*0*_ (+30 ± 16.6% on average; ES = 2.23 ± 0.28), reductions in *FV*_*imb*_ (-74.3 ± 54.7%; ES = 2.17 ± 0.27) and large increases in jump height (+12.4 ± 7.6%; ES = 1.45 ± 0.23). In this case, individual analysis showed that all subjects achieved improved jump height above the smallest worthwhile change, and reduced *FV*_*imb*_, as reported by Jiménez-Reyes et al (2017) [10], which supports and confirms the effectiveness of this kind of training approach. The time for individuals to reach their optimal F-v profile was 12.6 ± 4.6 weeks on average. When the results were split into two specific sub-groups according to the percentages and thresholds of *FV*_*imb*_ [10] they gave a better description of the adaptations, with a more specific response according to initial *FV*_*imb*_. For HFD and LFD, the specific heavy-load program resulted in extremely large increases in *F*_*0*_ (HFD: +44.1 ± 11.7% on average; ES = 3.24 ± 0.29; LFD: +16.5 ± 5.1% on average; ES = 3.00 ± 0.36), reductions in *FV*_*imb*_ (HFD: -118.2 ± 46.5% on average; ES = 5.27 ± 0.39; LFD: -31.1 ± 11.7% on average; ES = 3.09 ± 0.38) and large increases in jump height (HFD: +17.1 ± 8.1% on average; ES = 1.76 ± 0.33; LFD: +7.8 ± 2.8% on average; ES = 1.27 ± 0.17). The time needed to reach an optimal F-v profile ranged between 15.9 ± 3.8 weeks on average for HFD and 9.2 ± 2.0 weeks on average for LFD. Overall, these results are in line with those obtained by Jiménez-Reyes et al (2017) [10], but better in terms of jump performance, likely due to an individualized training duration to ensure the optimal profile was reached in participants at a higher competitive level. Thus, our results confirm the effectiveness and specificity of the exercises and loadings selected for this group for specifically shifting the F-v profile in accordance with an initial *FV*_*imb*_ showing a force-deficit (Table 2; Figs 1 and 2) [10]. These findings are also in agreement with other studies showing high-load training specificity [20,21,32,37,44]. The increase in *F*_*0*_ was observed here in parallel with a decrease in *v*_*0*_, even if no interrelationships can be supported between these two qualities, except that when one of these qualities was trained, the other was not. In the present study, the maximal strength improvement (*F*_*0*_) was not associated with the same kind of increase in *P*_*max*_, which would have been the case if subjects had kept their *v*_*0*_ value similar. Consequently, the performance improvement can be mainly attributed to *FV*_*imb*_ reduction, and less to an increase in *P*_*max*_, which justifies the interest in *FV*_*imb*_ in strength training focused on improving ballistic performance.

Finally, for this FD group, our results confirmed the speculation in our previous study about the required time (longer than the fixed time of 9 weeks) for eliciting adjustments at a structural level [18], as confirmed by times ranging between 15.9 ± 3.8 weeks on average for HFD and 9.2 ± 2.0 weeks on average for LFD sub-groups.

### Velocity-deficit sub-group–(HVD and LVD sub-groups)

In the VD group, the specific training caused moderate (as measured by jump height) to extremely large increases in *v*_*0*_ (+15.8 ± 5.1%; ES = 2.72 ± 0.29), reductions in *FV*_*imb*_ (-19.2 ± 6.9%; ES = 2.36 ± 0.35) and increases in jump height (+10.1 ± 2.7%; ES = 0.93 ± 0.09). These results are in line with the aforementioned pilot study [10], showing the effectiveness of this training approach in subjects with a velocity deficit (Tables 1 and 2; Figs 1 and 2). As in the FD group, similar results were found in terms of *FV*_*imb*_ reduction and jump height improvements, as in the original study. The slight difference observed in jump height improvements (+12.7 vs 10.1%) can be explained, as for the FD group, by the overall higher level of competition of the participants (more highly trained) in the current study. In the VD group, time to reach an optimal FV profile was 8.7 ± 2.1% weeks on average. When the results were split into two specific sub-groups according to the percentages and thresholds of *FV*_*imb*_ [10] the results showed a better description of adaptations with a more specific response according to initial *FV*_*imb*_, and for HVD and LVD, the specific “overspeed” exercises resulted in extremely large increases in *v*_*0*_ (HVD: +20.2 ± 2.4% on average; ES = 3.30 ± 0.18; LVD: +12.6 ± 3.9% on average; ES = 2.13 ± 0.29); reductions in *FV*_*imb*_ (HVD: -25.5 ± 2.4% on average; ES = 7.19 ± 0.52; LVD: -14.6 ± 5.3% on average; ES = 2.54 ± 0.15); and moderate increases in jump height (HVD: +11.6 ± 2.8% on average; ES = 1.12 ± 0.13; LVD: +9.1 ± 2.2% on average; ES = 0.78 ± 0.09). The time required to reach an optimal F-v profile ranged between 9.6 ± 1.9 weeks on average for HVD and 8.0 ± 2.1 weeks on average for LVD. For the whole VD group, the time required to reach an optimal F-v profile was very similar to the 9-week fixed-time schedule used in all programs in the original study.

As in the FD group, these results confirm, using a more individualized approach, the effectiveness and specificity of the exercises and loadings selected for this group for specifically shifting the F-v profile in accordance with initial *FV*_*imb*_ measurements showing a velocity deficit (Table 2; Figs 1 and 2), thus improving the maximal velocity end of the F-v relationship. These findings are also in agreement with other studies aiming at specifically improving velocity-related qualities [19,22,23,26], supporting the “principle of velocity specificity” as a specific stimulus to promote velocity-specific neural training adaptations [23,45–47]. As previously demonstrated [10], the main exercise used in the VD group was the “horizontal squat jump” [27], inducing an “overspeed” stimulus helping athletes to achieve lower limb extension velocities 20–30% higher than the take-off velocity of an SJ [22,27]. As in the FD sub-group, the increase in *v*_*0*_ in the VD group was observed in parallel with a decrease in *F*_*0*_, so following the same interpretation as above, the performance improvement can be mainly attributed to *FV*_*imb*_ reduction, and less to an increase in *P*_*max*_.

### Additional points

An interesting observation in the current study was that the time needed to reach the optimal F-v profile was significantly correlated to the initial *FV*_*imb*_ (r = 0.82, p<0.01), when considering all the participants or each subgroup (Fig 3). The larger the initial deficit, the longer the training duration necessary to reach the optimal profile. This may have practical value, since it may allow recommendations for the approximate duration of specific training programs depending on the initial *FV*_*imb*_. This may be related to training background and although all subjects were responders, it should be noted that there was variability within the HVD and HFD sub-groups.

Our findings support the value of a new step in the individualization of training and the need to individualize not only the training content but also the training duration. Including specific training duration as a parameter will provide more complete knowledge about effective training according to individual needs [9,10]. Given the ease of measurement throughout a season (limb extension, *P*_*max*_ and different jumps with few additional loads) [5,13] our recommendation is to monitor the evolution of *FV*_*imb*_ to decide when an athlete needs to change from one specific sub-group to another, adjusting training content, and with F-v monitoring, possibly also adjusting the training duration. This approach allows a dynamic adaptation in each individual’s response to training, in terms of both training content and timing. Keeping in mind the need to individualize due to the variability observed when a fixed time training period was used [10] and considering that some subjects adapt faster than others and may need to change sub-groups (e.g. from HFD to LFD) within the training period, intermediate assessments may allow easy fine-tuning of the training program and adaptation to the response kinetics of each individual. These intermediate assessments could be implemented every 1 to 3 weeks as we have performed in the present study, but it could be monitored much more frequently when the athlete is approaching the threshold since the approach is possible with only 2-loads [13], which make the assessment and decision quicker when necessary. The present results may provide valuable additional knowledge and potential applications in sport training practice, allowing more individualized, specific and effective training monitoring and periodization.

Another addition in this study was checking the changes in F-v profile parameters and jumping performance following training cessation after athletes reached their optimal profile. This point is of interest, mainly for team sports, since several physical qualities such as sprint performance, maximal strength and repeated sprint ability exhibit different kinetics during the tapering period that follows an intense training block [48,49]. In the case of lower limb maximal strength, although is not exactly the same variable as in our study, [48] reported that maximal strength could be maintained during a 3-week tapering period in highly trained rugby players. Although we cannot do a direct comparison since taper studies typically use an intensified training period before starting the tapering phase, it is reasonable that our protocol did not induce “performance rebound” because our athletes were following a specific and individualized progressive strength training until they reached their optimal F-v profile and then voluntarily refrained from strength training for 3 weeks while continuing with their specific sporting activities and competitions. During the detraining period in the present study, all the variables maintained their post-training values with only minimal changes (Table 1). This result may be very useful from a practical standpoint since in team sports, cessation of individualized strength training based on athletes’ F-v profiles could be a good strategy during taper periods or training camps preparing for major competitions, since the parameters related to the F-v profile and jumping performance are retained. Conversely, although we did not investigate longer de-training periods, we recommend monitoring the F-v variables every 3 weeks to decide whether a phase of specific training is necessary (in case of change in *FV*_*imb*_).

### Limitations

The main limitation was that we only considered the kinetics of detraining over a 3-week period. However, 3 weeks is common for the taper periods usually performed in team sports and this is the reason we decided to use this time-span. Also, it was not possible to continue with a prolonged detraining period during the in-season. Our aim was to reach the optimal F-v profile; once the participants reached this and completed the detraining period, they began a training program aiming to improve jump height and *P*_*max*_. By this point, all the subjects were in a well-balanced state and training needs changed related to F-v aspects.

## Conclusions

This study confirmed the results of the pilot investigation by Jiménez-Reyes et al [10], showing that an optimized and individualized training program specifically addressing the force-velocity imbalance is efficient at improving jumping performance even in trained subjects. *FV*_*imb*_ can therefore be considered as a potentially useful variable for prescribing optimal resistance training to improve ballistic (e.g. jumping) performance. The new information added by this study is that: (i) the high inter-subject variability in the timing of training-induced adaptations warrants regular monitoring of *FV*_*imb*_ over the training period, so that training content and duration is also individualised until the athlete reaches the targeted individual F-v profile; (ii) there is a positive correlation between the magnitude of individual *FV*_*imb*_ and the time necessary to reach optimal profile; and (iii) no significant changes in *FV*_*imb*_ or F-v profile variables and jump performance were observed in the 3-week detraining period studied. Collectively, these results provide useful insights into a more specific, individualised and accurate training prescription for jump height performance.

## Supporting information

S1 Dataset Original data. (XLSX)
